# A prospective multicenter register study exploring health-related quality of life in women with Sjögren's disease during pregnancy

**DOI:** 10.3389/fgwh.2026.1735104

**Published:** 2026-02-24

**Authors:** Maren Wisth Lie, Mina Nordli, Hege Suorza Svean Koksvik, Kjersti Grønning

**Affiliations:** 1 Department of Clinical and Molecular Medicine, Norwegian University of Science and Technology, Trondheim, Norway; 2 Department of Orthopaedics, Rheumatology and Dermatology, St Olav’s Hospital HF, Trondheim, Norway; 3 Department of Public Health and Nursing, Norwegian University of Science and Technology, Trondheim, Norway; 4 Department of Research, Levanger Hospital, Nord-Trøndelag Hospital Trust, Levanger, Norway

**Keywords:** antenatal care, health personnel, health-related quality of life (HRQoL), pregnancy, pregnancy outcome, Sjögren's disease

## Abstract

**Objectives:**

Limited research has investigated health-related quality of life (HRQoL) in women with Sjögren's syndrome. Pregnancies in women with Sjögren's disease are considered high-risk, with potential adverse outcomes for both the fetus and the mother. This study aims to explore HRQoL in women with Sjögren's disease during pregnancy.

**Methods:**

This prospective multicenter registry study explored data from pregnant women with Sjögren's syndrome enrolled in the nationwide quality registry, RevNatus. Data were collected from January 2016 to September 2023 and included demographic information, self-reported responses from the RAND-12 questionnaire assessing HRQoL, and visual analogue scales (VAS) evaluating pain, fatigue, and overall disease burden.

**Results:**

In total, 62 women with 75 pregnancies were included. These women scored highly across most domains related to HRQoL and reported low levels of pain and other disease-related symptoms. There were few to no significant differences in RAND-12 and VAS scores between the trimesters. However, a pattern emerged in the third trimester, characterized by lower exercise rates, increased work withdrawal, and higher disease activity. Most women had received counselling regarding their diagnosis and pregnancy, and the majority were in remission. Adverse outcomes, such as pre-eclampsia and premature birth, were not prominent.

**Conclusion:**

Norwegian women with Sjögren's disease generally report high and stable HRQoL during pregnancy. Their experiences of pain and disease activity are minimal. However, they face challenges related to physical functioning and maintaining regular exercise as pregnancy progresses, an area that should be targeted in antenatal care.

## Introduction

The World Health Organization (WHO) promotes a holistic approach to maternity care, emphasizing the individuality of each pregnancy (1). Although pregnancy and childbirth have been widely researched, evidence suggests a continued need for further investigation into women's reproductive and maternal health (2), especially since Sjögren's disease has been associated with increased risks of spontaneous abortion, preterm birth, low birth weight, and congenital anomalies in infants (3).

Primary Sjögren's disease is a chronic systemic autoimmune rheumatic disease marked by lymphoplasmacytic infiltration of the salivary and lacrimal glands, leading to sicca symptoms and/or systemic manifestations, often accompanied by a distinct autoantibody profile. It is the second most common connective tissue disease after rheumatoid arthritis, affecting 0.3%–3% of the population, with a strong female predominance (9:1 sex ratio) (4). Individuals with Sjögren's disease often experience fatigue and joint pain, with fatigue frequently emerging as a major and persistent concern (4). Both symptoms significantly impair patients' health-related quality of life (HRQoL) (4–6). Nevertheless, research shows that the consequences of Sjögren's disease vary among individuals, and the subjective experience of symptoms in daily life differs significantly (5).

Even though Sjögren's disease has been associated with adverse pregnancy outcomes (3), increased public awareness and improvements in early diagnosis and treatment of Sjögren's syndrome have contributed to a higher prevalence of pregnant women with Sjögren's disease (3, 7). Previous research on pregnancy and Sjögren's disease has primarily focused on adverse fetal and maternal outcomes, including low birth weight, preeclampsia, higher rates of stillbirth and abortion, and preterm delivery (3, 8). However, a recent study suggests that pregnancies in women with Sjögren's disease are no longer universally classified as high-risk. This shift is largely attributed to improved monitoring and care, despite the continued elevated risk of adverse fetal outcomes, such as congenital heart block in women who test positive for anti-SSA or anti-SSB antibodies (9). These patients are monitored more closely, with fetal heart assessments conducted between gestational weeks 16 and 24, supplemented by additional ultrasounds when necessary.

A different study (10) investigating the experiences and unmet needs of patients regarding pregnancy and family planning revealed that individuals with rare rheumatic diseases, such as Sjögren's disease, express a preference for a more holistic approach from healthcare providers, including gynecologists, midwives, and obstetricians. These patients favor care that places less emphasis on disease activity and more on overall well-being (10). Nevertheless, comprehensive preconception counselling and prenatal screening remain critical, as early identification of fetal abnormalities and strategic planning concerning the timing and method of delivery are essential for optimizing maternal and neonatal outcomes (3). In light of these considerations, the present study aims to explore changes in HRQoL and selected disease-specific parameters throughout pregnancy in Norwegian women diagnosed with Sjögren's disease.

## Materials and methods

### Design, sample, and data collection

This prospective multicenter cohort study analyses data from the nationwide quality registry RevNatus, which collects information from 19 of Norway's 22 rheumatology departments. Eligibility for inclusion in RevNatus are women over the age of 16 with an inflammatory rheumatic diagnosis confirmed by a rheumatologist and are either planning a pregnancy or currently pregnant. The registry gathers a broad range of variables, including self-reported health status, HRQoL measured by the RAND-12 (SF-12), pregnancy outcomes, disease activity indicators, medication use, comorbidities, and demographic data. RevNatus is designed to prospectively follow the women from the preconception phase through to 12 months postpartum. Data collection is conducted partly through self-reporting and partly by nurses or rheumatologists during scheduled outpatient consultations (11).

The inclusion criteria for this study comprise women diagnosed with primary Sjögren's disease who meet the American College of Rheumatology (ACR)/European League Against Rheumatism (EULAR) classification criteria (12) and are enrolled in the RevNatus registry. The RevNatus data was collected between January 2016 and September 2023. The first trimester consists of the first twelve weeks of pregnancy; the second trimester starts in week 13 and lasts until the end of week 28. The third and final trimester starts in week 29. Pregnancy outcome was collected six weeks after delivery and is registered as full-term (>37 weeks of gestation), pre-term (<37 weeks of gestation) or as abortion or stillbirth.

Demographic data including age, educational background, weight, height, physical activity, and employment status were self-reported. Age was categorized into three age groups: <30 years, 30–39 years, and 40–49 years. Body mass index (BMI) was calculated using self-reported weight and height from the first trimester and classified as underweight (<18.5 kg/m^2^), normal weight (18.5–24.9 kg/m^2^), overweight (25–29.9 kg/m^2^), or obese (>30 kg/m^2^). HRQoL was assessed using the RAND-12 questionnaire (SF-12). Participants rated their perceived quality of life by responding to 12 items using either a three- to five-point scale (e.g., Excellent, Very Good, Good, Fair, Poor) or closed-ended questions (e.g., Yes/No). The items cover eight domains: physical functioning (PF), bodily pain (BP), general health (GH), vitality (VT), social functioning (SF), role limitations due to emotional problems (RE), role limitations due to physical problems (RP), and mental health (MH). Each response is scored and transformed into a scale ranging from 0 to 100, with higher scores indicating better HRQoL. The RAND-12 is a widely used and validated generic instrument for assessing HRQoL (13, 14).

Visual analogue scales (VAS) were employed to assess patients' perceived disease burden (VAS-total), pain (VAS-pain), and fatigue (VAS-fatigue), which are commonly used metrics in rheumatological care for individuals with Sjögren's disease (15). Each VAS ranges from 0 to 100, with lower scores indicating minimal pain, fatigue, or disease burden, and higher scores reflecting greater severity. Clinicians also evaluated disease activity, categorizing patients into one of the following groups: disease debut, persistent disease, severe relapse, mild recurrent disease, remission, or unknown. Remission was defined as the absence of symptoms related to Sjögren's syndrome. Mild recurrent disease, debut, and severe relapse were considered active disease states, characterized by the presence of symptoms. Persistent disease referred to a prolonged period of low-grade activity with clinical improvement under treatment (16). Comorbidity data were collected by healthcare professionals. In addition, data were collected on pharmacological treatments and whether patients had received preconception counselling related to pregnancy and Sjögren's disease, categorized as Yes, No, or Uncertain.

### Data analysis

A descriptive analysis was conducted to provide an overview of the dataset (17). As most continuous variables were not normally distributed, results are presented as medians with interquartile ranges (IQR). Categorical variables are reported as frequencies and percentages, while selected descriptive variables are presented as means ± standard deviation. To examine differences in HRQoL, disease activity measures, physical activity, and employment status across trimesters, non-parametric Wilcoxon signed-rank tests were applied due to the non-normal distribution of the data. Although a few RAND-12 variables were approximately normally distributed in certain trimesters, this was not consistent across all time points; therefore, non-parametric tests were used for all variables to ensure comparability. A two-sided *p*-value ≤ 0.05 was considered statistically significant, and no adjustments were made for multiple comparisons. Statistical analyses were performed using IBM SPSS Statistics version 29.0.1.1 for Mac and Windows.

### Ethical considerations

The study received prior approval from the Regional Committee for Medical and Health Research Ethics (REK Midt 612998) and conducted in accordance with the local legislation and institutional requirements. The participants enrolled in the RevNatus registry provided written informed consent at the time of inclusion, either via paper form or through a secure digital platform. Before enrolment, each participant was thoroughly informed about the registry's purpose through both written documentation and verbal explanation, ensuring clarity and voluntary participation. The study was conducted in full accordance with the ethical principles outlined in the Declaration of Helsinki (18), including respect for individual autonomy, confidentiality, and the right to withdraw at any time without consequence.

## Results

### Sample characteristics

A total of 62 women were included in the study, accounting for 75 pregnancies. One participant had three registered pregnancies, while ten others had two each. At inclusion, the mean age of the sample was 31.8 ± 4.5 years, and the mean body mass index (BMI) was 23.6 ± 3.7 kg/m^2^. Most participants (77.3%) had completed higher education (college or university). Comorbidities were reported in 32% of the sample, with the most common being other connective tissue diseases (8%), followed by hypo- or hyperthyroidism (5.3%) and fibromyalgia (5.3%). Additional comorbidities included mental health disorders (4%), hypertension (4%), polycystic ovary syndrome (2.7%), ulcer disease (2.7%), psoriasis (2.7%), secondary antiphospholipid syndrome (1.3%), and uveitis (1.3%). No multiple gestation pregnancies were reported, and the majority (80%) were planned. Most participants (70.7%) received preconception counselling. Preeclampsia was documented in 4% of pregnancies, 1.3% developed HELLP syndrome and 86.7% were born at full term. Only one abortion/ stillbirth was registered. Most of the women (61.3%) used hydroxychloroquine at some point during pregnancy.

Descriptive data on employment status, physical activity, and clinical measures across the trimesters are summarized in Table 1. Most participants were classified as being in remission by their rheumatologist, and disease activity remained relatively stable throughout pregnancy, with no statistically significant differences observed between trimesters. In contrast, employment status showed statistically significant changes across all three trimesters (T1–T2, *p* = 0.006; T1–T3, *p* < 0.001; T2–T3, *p* < 0.001). At the beginning of pregnancy, 85.7% of participants were employed either full- or part-time, with a progressive decline in employment by the third trimester. A statistically significant difference between the second and third trimesters in physical activity/exercise (*p* = 0.037) was also registered, with less physical activity in the third trimester.

**Table 1:** Trimester characteristics (*n* = pregnancies).

Variables	1. trimester		2. trimester		3. trimester	
	*n* = 49	*n* (%)	*n* = 74	*n* (%)	*n* = 67	*n* (%)
Employment						
Full-time employment	33	(67.3)	36	(48.6)	19	(28.4)
Part-time employment	9	(18.4)	16	(21.6)	12	(17.9)
Not working, disease related	3	(6.1)	14	(18.9)	19	(28.4)
Not working, other reason	2	(4.1)	3	(4.1)	7	(10.4)
Student	1	(2.0)	1	(1.4)	0	
Parental benefit	0		1	(1.4)	8	(11.9)
Pregnancy allowance	1	(2.0)	2	(2.7)	1	(1.5)
Missing	0		1	(1.4)	1	(1.5)
Physical activity/exercise						
3 or more times a week	5	(10.2)	6	(8.1)	2	(3.0)
1–2 times a week	12	(24.5)	16	(21.6)	11	(16.4)
1–3 times a month	4	(8.2)	0		4	(6.0)
Not regularly	18	(36.7)	31	(41.9)	31	(46.3)
Missing	10	(20.4)	21	(28.4)	19	(28.4)
Clinical measures						
Disease activity						
Remission	10	(20.4)	38	(51.4)	41	(61.2)
Mild recurrent disease	0		1	(1.4)	2	(3.0)
Persistent disease	1	(2.0)	8	(10.8)	9	(13.4)
Unknown	0		3	(4.0)	5	(7.5)
Missing	38	(77.6)	24	(32.4)	10	(14.9)
	**Mean**	**SD**	**Mean**	**SD**	**Mean**	**SD**
CRP in mg/L	4.7	(9.3)	4.8	(6.2)	5.5	(9.6)

SD, standard deviation.

### HRQoL and disease-related measures during pregnancy

Overall, there are limited variations within the trimesters for the RAND12-variables (Table 2). PF is the only sub-scale subsequent to change negatively from the first- to the third trimester. RP has the lowest overall score in all trimesters followed by VT. The median scores for PF, RE, MH, BP and SF are all relatively high. The analyses revealed statistically significant differences between the first- and second trimesters for the PF domain (*p* = 0.004) and between the first- and third trimesters (*p* = 0.002). For the RE domain, statistically significant differences were only found between the second- and third trimesters (*p* = 0.015). For the BP domain, there was a statistically significant difference when comparing the first- and second trimester (*p* = 0.021), and the first- and third trimester (*p* = 0.032). The remaining RAND12 domains presented in Table 2 yielded no statistically significant differences between the trimesters.

**Table 2:** Changes in HRQoL and disease-related measures during pregnancy (*n* = pregnancies).

Variables	T1	T2	T3	Changes from T1–T2	Changes from T1–T3	Changes from T2–T3
	*n* = 49	*n* = 74	*n* = 67			
	Median (IQR)	Median (IQR)	Median (IQR)	*P*-value	*P*-value	*P*-value
HRQoL						
RAND12						
Physical Functioning (PF)	100 (25)	50 (75)	50 (63)	**0** **.** **004**	**0** **.** **002**	0.125
Role Physical (RP)	0 (100)	0 (63)	0 (50)	0.821	0.149	0.302
Role Emotional (RE)	100 (0)	100 (50)	100 (0)	0.473	0.482	**0** **.** **015**
Mental Health (MH)	70 (20)	70 (20)	70 (20)	0.957	0.664	0.202
Bodily Pain (BP)	75 (31)	75 (25)	75 (50)	**0** **.** **021**	**0** **.** **032**	0.855
General Health (GH)	50 (50)	50 (50)	50 (50)	0.882	0.516	0.499
Vitality (VT)	20 (40)	20 (40)	40 (20)	0.710	0.477	0.735
Social Functioning (SF)	75 (50)	75 (25)	75 (50)	0.282	0.564	0.101
Disease-related measures						
VAS-scales						
VAS-pain	20 (29)	20 (40)	29 (45)	0.270	0.122	0.676
VAS-fatigue	65.50 (40)	60 (55)	55 (34)	**0** **.** **046**	0.087	0.750
VAS-total	26 (39)	45 (46)	40 (41)	0.935	0.754	0.678

Wilcox signed rank test analyses *p*-value ≤ 0.05. T1 = 1. Trimester, T2 = 2. Trimester, T3 = 3. Trimester. IQR, interquartile range.

Bold values are *P*-value ≤ 0.05.

For the VAS-scales (pain, fatigue and total) the scores were relatively stable throughout pregnancy (Table 2). The analysis revealed relatively low scores throughout pregnancy for VAS-pain and VAS-total. VAS-fatigue was relatively high in the first trimester but decreased in the second trimester (*p* = 0.046).

## Discussion

This study aimed to explore HRQoL in Norwegian women with Sjögren's disease throughout pregnancy to detect areas where women with Sjögren's disease could need more guidance from midwives and health care personnel providing antenatal care. In general, the analysis showed limited variations in HRQoL and disease-related measures during pregnancy, indicating that the pregnant women with Sjögren's disease in this study experienced little pain and perceive overall good health.

The findings in this study are in contrasts with previous research on HRQoL in individuals with Sjögren's disease (4, 5, 19, 20) stating that Sjögren's disease has a high impact on social and physical dimensions of patients' HRQoL. Previous research has also stated that people with Sjögren's disease experience challenges related to social and emotional dimensions (19, 20). In this study, the pregnant women with Sjögren's disease had lower scores for the RAND12 dimensions vitality, entailing limitations relating to energy and tiredness, the role-physical dimension, encompassing limitations in daily activities involving social and work-related situations, and general health, which is known to reflect on the individual's psychological wellbeing (21). Moreover, the RAND-12 dimensions of role-emotional and social functioning remain consistently high and stable within this study sample, reflecting limited interference of depressive and anxious symptoms with daily activities, as well as minimal disruption of social engagement due to emotional or physical health issues. The consistently high HRQoL scores observed in this cohort, despite Sjögren's disease being associated with substantial disease burden in previous studies (19, 20), may reflect several protective factors. Most participants were in remission during pregnancy, which likely minimized symptom severity and reduced the impact of disease activity on daily functioning (9, 22). Also, the majority had received comprehensive preconception counselling and planned their pregnancies, enabling optimization of health status before conception and adherence to preventive strategies such as medication management and monitoring (22, 23). Additionally, most had completed higher education (college/university), a factor known to positively correlate with the adoption of healthy lifestyle behaviors and the capacity to engage with health-related information and preventive care strategies (24). This educational background may have facilitated access to and comprehension of preconception counselling, as shown by the participants' adherence to recommendations such as pregnancy planning and achieving disease remission prior to conception (8, 22). Furthermore, higher educational background may have enhanced their ability to seek and utilize preventive measures during pregnancy, thereby contributing to safer and healthier pregnancy outcomes (22, 24).

Moreover, the physiological changes of pregnancy may exert a modifying effect on autoimmune disease activity, as suggested by improvements reported in other rheumatic conditions during gestation (23). In addition, Norway's healthcare system, characterized by structured antenatal care and access to multidisciplinary support, has likely contributed to enhanced maternal well-being. Taken together, these factors provide a plausible explanation for the consistently high and stable HRQoL observed in this cohort, contrasting with previous literature that highlights significant physical and psychosocial impairment among individuals with Sjögren's disease (4, 6, 19, 20).

The VAS-pain and bodily pain scores show that this study sample reports low amounts of pain during pregnancy, which is contrary to research portraying pain as one of the primary clinical manifestations for people with Sjögren's disease (4, 6, 19). Interestingly, symptoms of other rheumatologic diseases, such as RA, have been seen to spontaneously improve during pregnancy (23), and previous research has implicated symptom overlap between Sjögren's disease and RA (4). On the other hand, Sjögren's disease also present with similar symptoms as those commonly found in people with systemic lupus erythematosus (SLE) (4). However, this study did not take into account variables related to disease activity scores such as anti-SSA and anti- SSB levels, which are known factors to influence adverse maternal and fetal outcomes as well as disease exacerbations in people with Sjögren's disease (3, 8). This study focused exclusively on HRQoL, perceived disease burden, pain, and fatigue in pregnant women with Sjögren's disease. The findings indicate that levels of pain, fatigue, and overall perceived disease burden remained relatively stable throughout the course of pregnancy in this study population. However, fatigue was relatively high in the first trimester but decreased in the second trimester. Yet statistically significant differences were only seen when comparing the first and second trimesters. Our findings of relatively high VAS-fatigue scores and low scores in the vitality domain of HRQoL, indicate that fatigue is a symptom of great significance and align with previous research that fatigue is a major problem for patients with Sjögren's disease (4, 5, 20, 25, 26). The elevated VAS fatigue scores observed in the first trimester are not unexpected, given that fatigue and tiredness are common and impactful symptoms even among healthy pregnant women (27). This suggests that the reported fatigue may, at least in part, reflect normal physiological changes associated with pregnancy rather than disease-specific manifestations.

The majority of participants in this study remained in full- or part-time employment until the third trimester, which contrasts with findings from previous research (19) indicating lower rates of paid employment among individuals with Sjögren's disease. On the other hand, role-physical, a domain related to limitations in various roles, such as daily activities and work (21), had the lowest overall score in the study. A large Norwegian longitudinal study also found fatigue/tiredness to be one of the main factors influencing the rate of sick leave in pregnant women (28). It is therefore noteworthy that the women in this study continued working with relatively low rates of sick leave, particularly during the first trimester, despite reporting high levels of fatigue on the VAS and low scores on vitality and role-physical domains. These findings diverge from those reported by Dørheim et al. (28), who observed greater functional impairment in similar populations. Thus, the rate of sick leave among pregnant women has increased in recent years (29). In this study, a similar trend was observed, with a growing proportion of participants not engaged in paid employment as pregnancy progressed toward the third trimester. However, elevated sick leave rates are also common in the general pregnant population (28), suggesting that pregnancy itself may be a contributing factor to work withdrawal, independent of underlying disease.

This study also shows that this cohort of women with Sjögren's disease report relatively high HRQoL for most RAND-12 domains and have few disease complaints during pregnancy. The women were physically active in the first trimester, as advised in guidelines for pregnant women with connective tissue diseases (22). A decline in the RAND-12 physical functioning domain was reported toward the third trimester, suggesting that physical functioning may warrant greater attention in antenatal care for women with Sjögren's disease. This finding aligns with previous research indicating that impaired physical functioning significantly contributes to reduced HRQoL in individuals with Sjögren's disease (19). Moreover, this study found higher rates of exercise in the first trimester, confirming findings from another study indicating that the level of exercise is prone to decrease when women become pregnant (30). The elevated fatigue scores in the first trimester and the decline in physical functioning and exercise levels toward the third trimester found in this study mirror patterns reported in healthy pregnant populations (28, 31, 32) where physiological changes due to pregnancy may contribute to tiredness and decreased activity. Similarly, the reduction in physical activity during late pregnancy aligns with research showing that exercise levels typically decrease as pregnancy progresses in the general population (31). These parallels suggest that some symptoms traditionally attributed to Sjögren's disease during pregnancy (8) may, in fact, reflect normal gestational changes (33) rather than disease-specific manifestations (34). However, the consistently high HRQoL scores and low pain levels observed in this cohort contrast with previous literature on Sjögren's disease outside pregnancy, which reports significant negative impacts on physical and social functioning (7). The declining frequency of exercise after the first trimester could be a focus area for antenatal care since physical activity is considered especially important for women with connective tissue diseases (22). Exercise is shown to be beneficial for managing fatigue and pain for women with Sjögren's disease (4) and may improve patients' HRQoL (6, 35).

Previous studies have found it difficult to conclude on adverse maternal and fetal outcomes for women with Sjögren's disease due to difficulties in dealing with confounding factors such as BMI, age and comorbidities (3, 34). In this study, the rate of overweight was lower (14.7%) than in the general Norwegian pregnant population (22%) (36). The majority were also considered to be normal weight at the beginning of pregnancy, as recommended by the Norwegian Institute of Public Health (FHI) (29). The mean age of the study population was slightly higher (31.8 ± 4.5 years) when compared to the general pregnant population (31.4 years) for all births in Norway (29), but the majority were not considered to be of advanced maternal age (37). Although the majority of participants did not have registered comorbidities, the proportion with documented comorbidity (32%) was notably higher than that reported in the general pregnant population in Norway (15.3%) (38). It is therefore unexpected that these women demonstrated high scores across most RAND-12 domains and exhibited limited variation in HRQoL, given that recent literature has identified comorbidities as significant contributors to diminished quality of life (39).

In this study, 86.7% of participants carried out their pregnancies full term, with only 1.3% resulting in stillbirth or abortion. These outcomes contrast with previous research, which has reported higher rates of adverse fetal outcomes, including preterm birth, stillbirth, and reduced live birth rates among women with Sjögren's disease (3, 4, 8, 34, 40). Additionally, the observed rate of preeclampsia was 4%, lower than that reported in a recent review (8). This discrepancy may be attributable to the implementation of preventive strategies, such as the use of acetylsalicylic acid (ASA) and enhanced prenatal monitoring for women with Sjögren's disease in Norway (22).

### Strengths and limitations

One of the major strengths of this study is the RevNatus register data, enabling the exploration of a rare disease such as Sjögren's disease, where recruitment could be difficult. Additionally, the register has nationwide coverage in Norway, and the prospective data benefits from greater accuracy as the variables are assessed by trained professionals (41). On the other hand, managing and registering in a quality register is challenging (11) and several women were not included by their rheumatologist until they were already pregnant, resulting in missing data from some pregnancies and subsequently a smaller sample size. The relatively small sample size and conducting multiple comparisons across several HRQoL domains and trimesters increases the risk of type I error. The findings should therefore be interpreted with care. A limitation of this study is the absence of a healthy pregnancy control group. While the findings suggest that women with Sjögren's disease report high and stable HRQoL and minimal disease burden during pregnancy, the lack of direct comparison with healthy pregnant women makes it difficult to determine whether these outcomes are unique to Sjögren's disease or simply reflect normal physiological changes associated with pregnancy. Future research should include a matched control group of healthy pregnant women to clarify whether observed patterns are disease-specific or part of the typical pregnancy trajectory. Additionally, longitudinal studies incorporating objective disease activity markers (e.g., ESSDAI) could help identify potential interactions between pregnancy and disease activity.

## Conclusion

This study found that the cohort of women with Sjögren's disease maintained high and stable HRQoL throughout pregnancy. Contributing factors may include high rates of preconception counseling, good overall health status, higher educational attainment, and a predominance of planned pregnancies. Participants also reported relatively low levels of pain and perceived disease burden. These findings are clinically relevant for midwives, obstetricians, and other healthcare professionals involved in antenatal care, as they emphasize the importance of tailored, person-centered approaches to support women with Sjögren's disease during pregnancy. Furthermore, the results highlight the importance of preconception counseling, structured pregnancy planning, and strategies that promote physical activity and fatigue management, as these may contribute to improved maternal well-being and pregnancy outcomes.

## Data Availability

The data that supports the findings of this study are available from the corresponding author upon reasonable request.

## References

[1] WHO. Maternal health Available online at: https://www.who.int/: World Health Organization (2024). Available online at: https://www.who.int/health-topics/maternal-health#tab=tab_1 (Accessed October 29, 2025).

[2] Souza JP Day LT Rezende-Gomes AC Zhang J Mori R Baguiya A A global analysis of the determinants of maternal health and transitions in maternal mortality. Lancet Glob Health. (2024) 12(2):e306–e16. 10.1016/S2214-109X(23)00468-0 38070536

[3] Geng B Zhang K Huang X Chen Y. A meta-analysis of the effect of Sjögren’s syndrome on adverse pregnancy outcomes. Clinics (Sao Paulo). (2022) 77:100140. 10.1016/j.clinsp.2022.100140 36403428 PMC9678673

[4] Parisis D Chivasso C Perret J Soyfoo MS Delporte C. Current state of knowledge on primary Sjögren’s syndrome, an autoimmune exocrinopathy. J Clin Med. (2020) 9(7):61. 10.3390/jcm9072299 PMC7408693 32698400

[5] Perella C Steenackers M Robbins B Stone L Gervais R Schmidt T Patient experience of Sjögren’s disease and its multifaceted impact on patients’ lives. Rheumatol Ther. (2023) 10(3):601–14. 10.1007/s40744-023-00531-7 36797434 PMC10140221

[6] Liu Z Dong Z Liang X Liu J Xuan L Wang J Health-related quality of life and psychological status of women with primary Sjögren’s syndrome: a cross-sectional study of 304 Chinese patients. Medicine (Baltimore). (2017) 96(50):e9208. 10.1097/MD.0000000000009208 29390343 PMC5815755

[7] Bayetto K Logan R. Sjögren’s syndrome: a review of aetiology, pathogenesis, diagnosis and management. Aust Dent J. (2010) 55(s1):39–47. 10.1111/j.1834-7819.2010.01197.x 20553243

[8] Yang Y Huang X-X Huo R-X Lin J-Y. Impact of Sjögren’s syndrome on maternal and fetal outcomes following pregnancy: a systematic review and meta-analysis of studies published between years 2007–2022. Arch Gynecol Obstet. (2024) 309(4):1135–49. 10.1007/s00404-023-07259-3 37921880

[9] de Frémont GM Costedoat-Chalumeau N Lazaro E Belkhir R Guettrot-Imbert G Morel N Pregnancy outcomes in women with primary Sjögren’s syndrome: an analysis of data from the multicentre, prospective, GR2 study. Lancet Rheumatol. (2023) 5(6):e330–e40. 10.1016/S2665-9913(23)00099-1 38251600

[10] Marinello D Zucchi D Palla I Aguilera S Galetti I Holmner M Exploring patient’s experience and unmet needs on pregnancy and family planning in rare and complex connective tissue diseases: a narrative medicine approach. RMD Open. (2022) 8(2):e002643. 10.1136/rmdopen-2022-002643 36597980 PMC9748988

[11] Bjørngaard H Koksvik HS Jakobsen B Grønning K. Nurses experience increased clinical and organisational competence by working with a medical quality register, RevNatus—a qualitative study. BMC Health Serv Res. (2022) 22(1):1291. 10.1186/s12913-022-08595-x 36289511 PMC9608925

[12] Shiboski CH Shiboski SC Seror R Criswell LA Labetoulle M Lietman TM 2016 American college of rheumatology/European league against rheumatism classification criteria for primary Sjögren’s syndrome: a consensus and data-driven methodology involving three international patient cohorts. Arthritis Rheumatol. (2017) 69(1):35–45. 10.1002/art.39859 27785888 PMC5650478

[13] Andersen JR Breivik K Engelund IE Iversen MM Kirkeleit J Norekvål TM Correlated physical and mental health composite scores for the RAND-36 and RAND-12 health surveys: can we keep them simple? Health Qual Life Outcomes. (2022) 20(1):89. 10.1186/s12955-022-01992-0 35659237 PMC9166415

[14] Ware JE Kosinski M Keller SD. A 12-item short-form health survey: construction of scales and preliminary tests of reliability and validity. Med Care. (1996) 34(3):220–33. 10.1097/00005650-199603000-00003 8628042

[15] Seror R Baron G Camus M Cornec D Perrodeau E Bowman SJ Development and preliminary validation of the Sjögren’s tool for assessing response (STAR): a consensual composite score for assessing treatment effect in primary Sjögren’s syndrome. Ann Rheum Dis. (2022) 81(7):979–89. 10.1136/annrheumdis-2021-222054 35393271 PMC9209686

[16] Stone JH Hoffman GS Merkel PA Min YI Uhlfelder ML Hellmann DB A disease-specific activity index for Wegener’s granulomatosis: modification of the Birmingham vasculitis activity score. International network for the study of the systemic vasculitides (INSSYS). Arthritis Rheum. (2001) 44(4):912–20. 10.1002/1529-0131(200104)44:4<912::AID-ANR148>3.0.CO;2-5 11318006

[17] Polit DF. Statistics and Data Analysis for Nursing Research. 2nd ed Upper Saddle River, N.J: Pearson (2010).

[18] Association WM. World medical association declaration of Helsinki: ethical principles for medical research involving human participants. Jama. (2025) 333(1):71–4. 10.1001/jama.2024.21972 39425955

[19] Dias LH Miyamoto ST Giovelli RA de Magalhães CIM Valim V. Pain and fatigue are predictors of quality of life in primary Sjögren’s syndrome. Adv Rheumatol. (2021) 61(1):28. 10.1186/s42358-021-00181-9 34051867

[20] Bowman SJ Seror R Porcher R Arends S de Wolff L Verstappen G Primary Sjögren’s disease: a review of unmet need, outcome measures, therapeutic advances and health economic impacts. Lessons from the new clinical endpoints in primary Sjögren’s syndrome: an interventional trial based on stratifYing patients (NECESSITY) innovative health initiative (IHI). Ann Rheum Dis. (2025) 84(7):1068–89. 10.1016/j.ard.2025.05.004 40527714

[21] Ware J Ma K Keller SD. SF-36 Physical and Mental Health Summary Scales: A User’s Manual. Boston, MA: Health Assesment Lab (1994).

[22] Wallenius M Skorpen CG Gjerdalen G Salvesen KÅ Grindheim S. Revmatisk Inflammatorisk Sykdom (Artrittsykdommer, Bindevevssykdommer og Vaskulitter) [Rheumatic Inflammatory Disease (Arthritic Diseases, Connective Tissue Diseases and Vasculitis)]: Norsk Gynekologisk Forening (2020). Available online at: https://www.legeforeningen.no/foreningsledd/fagmed/norsk-gynekologisk-forening/veiledere/veileder-i-fodselshjelp/revmatisk-inflammatorisk-sykdom (Accessed February 17, 2020).

[23] Giles I Yee C-S Gordon C. Stratifying management of rheumatic disease for pregnancy and breastfeeding. Nat Rev Rheumatol. (2019) 15(7):391–402. 10.1038/s41584-019-0240-8 31186540

[24] Raghupathi V Raghupathi W. The influence of education on health: an empirical assessment of OECD countries for the period 1995–2015. Arch Public Health. (2020) 78:20. 10.1186/s13690-020-00402-5 32280462 PMC7133023

[25] Mæland E Miyamoto ST Hammenfors D Valim V Jonsson MV. Understanding fatigue in Sjögren’s syndrome: outcome measures, biomarkers and possible interventions. Front Immunol. (2021) 12:703079. 10.3389/fimmu.2021.703079 34249008 PMC8267792

[26] Rojas-Alcayaga G Herrera A Espinoza I Rios-Erazo M Aguilar J Leiva L Illness experience and quality of life in Sjögren syndrome patients. Int J Environ Res Public Health. (2022) 19(17):10969. 10.3390/ijerph191710969 36078685 PMC9518497

[27] Bai G Korfage IJ Groen EH Jaddoe VW Mautner E Raat H. Associations between nausea, vomiting, fatigue and health-related quality of life of women in early pregnancy: the generation R study. PLoS One. (2016) 11(11):e0166133. 10.1371/journal.pone.0166133 27814390 PMC5096665

[28] Dørheim SK Bjorvatn B Eberhard-Gran M. Sick leave during pregnancy: a longitudinal study of rates and risk factors in a Norwegian population. Bjog. (2013) 120(5):521–30. 10.1111/1471-0528.12035 23130975

[29] Macsali F. Health During Pregnancy and Childbirth Oslo: Norwegian Institute of Public Health (FHI) (2018). Available online at: https://www.fhi.no/nettpub/hin/grupper/svangerskap/ (Accessed February 1, 2023).

[30] Sitzberger C Hansl J Felberbaum R Brössner A Oberhoffer-Fritz R Wacker-Gussmann A. Physical activity in high-risk pregnancies. J Clin Med. (2022) 11(3):703. 10.3390/jcm11030703 35160151 PMC8836910

[31] Bø K Artal R Barakat R Brown W Davies GA Dooley M Exercise and pregnancy in recreational and elite athletes: 2016 evidence summary from the IOC expert group meeting, Lausanne. Part 1-exercise in women planning pregnancy and those who are pregnant. Br J Sports Med. (2016) 50(10):571–89. 10.1136/bjsports-2016-096218 27127296

[32] Jiang H Qian X Li M Lynn H Fan Y Jiang H Can physical activity reduce excessive gestational weight gain? Findings from a Chinese urban pregnant women cohort study. Int J Behav Nutr Phys Act. (2012) 9:12. 10.1186/1479-5868-9-12 22321640 PMC3306269

[33] Sedov ID Cameron EE Madigan S Tomfohr-Madsen LM. Sleep quality during pregnancy: a meta-analysis. Sleep Med Rev. (2018) 38:168–76. 10.1016/j.smrv.2017.06.005 28866020

[34] Ballester C Grobost V Roblot P Pourrat O Pierre F Laurichesse-Delmas H Pregnancy and primary Sjögren’s syndrome: management and outcomes in a multicentre retrospective study of 54 pregnancies. Scand J Rheumatol. (2017) 46(1):56–63. 10.3109/03009742.2016.1158312 27191226

[35] Nacar NE Karaborklu Argut S Unal E. Effects of exercise in primary Sjögren’s syndrome: a systematic review and meta-analysis of randomized clinical trials. Disabil Rehabil. (2025) 47(20):5196–204. 10.1080/09638288.2025.2474085 40062666

[36] Surén P Gunnes N Roth C Bresnahan M Hornig M Hirtz D Parental obesity and risk of autism spectrum disorder. Pediatrics. (2014) 133(5):e1128–e38. 10.1542/peds.2013-3664 24709932 PMC4006442

[37] Frederiksen LE Ernst A Brix N Braskhøj Lauridsen LL Roos L Ramlau-Hansen CH Risk of adverse pregnancy outcomes at advanced maternal age. Obstet Gynecol. (2018) 131(3):457–63. 10.1097/AOG.0000000000002504 29420406

[38] Örtqvist AK Magnus MC Söderling J Oakley L Nybo Andersen A-M Håberg SE The association between maternal characteristics and SARS-CoV-2 in pregnancy: a population-based registry study in Sweden and Norway. Sci Rep. (2022) 12(1):8355. 10.1038/s41598-022-12395-y 35589871 PMC9120467

[39] Sukumaran L Winston A Sabin CA. Understanding the conditions included in data-driven patterns of multimorbidity: a scoping review. Eur J Public Health. (2024) 34(1):35–43. 10.1093/eurpub/ckad179 37837614 PMC10843942

[40] Chan TM Wu CE Yu HH Hsiao CY Su TH Chen CB Fetal-neonatal and maternal outcomes in women with Sjögren syndrome: a population-based registry linkage study. Rheumatology (United Kingdom). (2023) 62(8):2820–8. 10.1093/rheumatology/keac711 36610986

[41] Andrade C. Research design: cohort studies. Indian J Psychol Med. (2022) 44(2):189–91. 10.1177/02537176211073764 35655982 PMC9120971

